# Treatment with anti-SARS-CoV-2 monoclonal antibodies in pregnant and postpartum women: first experiences in Florence, Italy

**DOI:** 10.1007/s15010-022-01777-z

**Published:** 2022-03-07

**Authors:** Tommaso Manciulli, Giulia Modi, Irene Campolmi, Beatrice Borchi, Michele Trotta, Michele Spinicci, Filippo Lagi, Alessandro Bartoloni, Lorenzo Zammarchi

**Affiliations:** 1 Department of Experimental and Clinical Medicine, University of Florence, Largo Brambilla 3, Largo Giovanni Alessandro Brambilla, 3, 50134 Florence, FI Italy; 2 Infectious and Tropical Diseases Unit, Careggi University Hospital, Florence, Italy; 3 Referral Centre for Infectious Diseases in Pregnancy, Careggi University Hospital, Florence, Italy

**Keywords:** SARS-CoV-2, Pregnancy, mAbs, Monoclonal antibodies, Casirivimab/imdevimab, COVID-19

## Abstract

**Purpose:**

Pregnant and postpartum women are at increased risk of developing severe COVID-19. Monoclonal antibodies (mAbs) are now widely used in high-income countries to treat mild to moderate COVID-19 outpatients at risk for developing severe disease. Very few data are available on the use of mAbs in special populations, including pregnant and postpartum women. Here we present our early experience with mAbs in these two populations.

**Methods:**

Electronic records of pregnant and postpartum women treated with mAbs at Careggi University Hospital, Florence, were retrieved. Relevant data were extracted (age, presence of risk factors for COVID-19, oxygen support, mAb type, gestational age, and pregnancy status). When available, outcomes at 28 days after administration were also included.

**Results:**

From March 1st to September 30th 2021, eight pregnant and two postpartum women have been treated with mAbs at our center. The median age was 31 years (IQR 30–33.5, range 29–38), median gestational age was 24 weeks. Seven patients had additional risk factors. According to the Italian disposition, all patients received casirivimab/imdevimab, with five receiving a 2.4 mg dose and five receiving a 8 g dose. Eight patients improved. One developed myocarditis, considered a COVID-19 complication. Another required a transient increase of low flow oxygen support before improving and being discharged. At a 28 days follow-up, all patients were clinically recovered. We did not observe mAbs related adverse events.

**Conclusion:**

Although preliminary data should be interpreted with caution, it is remarkable how mAbs were well tolerated by pregnant women with COVID-19. Further data on mAbs in this special population should be collected but the use of mAbs in pregnant and postpartum patients should be considered. Even thus oral antivirals are becoming available, they are not recommended in pregnant and postpartum women. This population may specifically benefit from treatment with last generation mAbs.

## Background

For about 2 years, the world has dealt with the severe acute respiratory syndrome coronavirus (SARS-CoV)-2 pandemic. While it was immediately clear that older adults with comorbidities had an increased risk for mortality and severe disease manifestations [1–3], pregnant and postpartum women were later recognized to be at higher risk for developing severe disease and poor outcomes [4–6] especially if the infection occurs in the third trimester [7]. In particular, a systematic review found that pregnant women have a higher odds of death (2.58) and of ICU admission (18.58) and babies born from pregnant COVID-19 patients also have a higher odd for neonatal ICU admission (4.58) [6]. The Centers for Disease Control and Prevention (CDC) listed pregnancy among the medical conditions associated with higher risk for severe COVID-19 [3].

Pregnant women were initially excluded from COVID-19 vaccination campaigns due to the absence of safety and efficacy data on this subset of patients [8, 9]. However, since mid-2021, several scientific societies have recommended vaccination of pregnant women against COVID-19 [8]. The relative delay and the fact that some pregnant women may feel hesitant about being vaccinated, put many pregnant women at risk for being infected with SARS-CoV-2.

From March 2021, monoclonal antibodies (mAbs) became available to treat COVID-19 in Italy [10]. At first, these were used only in outpatients with mild to moderate COVID-19, with risk factors for developing severe disease, and within 10 days from symptoms onset [11]. Then, recent data has shown a potential benefit in inpatients hospitalized for COVID-19 [12]. For this reason, since August 2021, in Italy the Agenzia Italiana del Farmaco (AIFA) has authorized the use of a higher dose of mAbs (casirivimab/imdevimab 8 g) in hospitalized seronegative patients with COVID-19, not requiring high flow oxygen or mechanical ventilation [13]. Likewise, the National Health Institute (NIH) guidelines state that the therapeutic management of pregnant patients with COVID-19 should be the same as for nonpregnant patients and anti-SARS-CoV-2 mAbs can be considered for pregnant people with COVID-19, especially those who have additional risk factors for severe disease [14].

AIFA includes “primary and secondary immunosuppression conditions” as risk factors for developing severe COVID-19 and eligibility criteria for mAbs prescription in outpatient subjects [11]. Considered the immune alterations associated with pregnancy and puerperium [15], the increasing evidence on poorer COVID-19 outcome in pregnant women [9, 16–21] and the recommendations issued by the National Institute of Health (NIH) [14] as well as the Royal College of Obstetrics and Gynecologists [22], in our center we offered mAbs to all pregnant women with mild to moderate COVID-19, not requiring hospitalization. Nevertheless, the use of mAbs in pregnant women is still scarcely documented in the medical literature. Here, we report the early results on the use of mAb to treat pregnant or postpartum patients at a single center in Italy.

## Methods

### Inclusion criteria

Electronic records of pregnant patients treated with mAbs from March 1st 2020 to September 30th 2021 at the Infectious and Tropical Diseases Unit, Careggi University Hospital, Florence, Italy, were retrieved. We included any pregnant or postpartum woman treated with either casirivimab/imdevimab 2.4 g (patients not hospitalized for COVID-19) or casirivimab/imdevimab 8 g (patients hospitalized for COVID-19). Patients treated at our outpatient service were sent by general physicians or other territorial medical units dedicated to the follow-up of COVID-19 patients at home. Women admitted to the hospital were treated in different Units of the Careggi University Hospital, Florence, Italy.

### Data collection

Data were captured from the electronic records, including age and gestational age at the time of admission, patient class (inpatient or outpatient), pregnancy class (pregnant or postpartum at administration), gestational outcome (concluded pregnancy, complicated delivery, pregnancy in progress), NIH severity scale at entrance [14], presence of adverse reaction to mAbs administration, Body Mass Index (BMI), presence of comorbidities. We also collected data on therapeutic strategies used to treat COVID-19 in inpatients.

Data were collected in an Excel spreadsheet (Microsoft, Redmond, CA). Categorical variables are presented as mean and percentages, continuous variables are presented as medians and interquartile ranges. In accordance with the procedure set by the AIFA, patients have been called back after 28 days for a telephonic follow-up.

## Results

### Demographics

A total of 10 patients were treated with mAbs at our hospital in the study period. Two patients were managed in the outpatient clinic, whereas 8 patients were hospitalized (inpatients). The median age was 31 years (IQR 30–33.5, range 29–38). At the time of admission, the mean gestational age was 24 weeks (IQR 21.5–25, range 17–38). Eight were treated during pregnancy and two, admitted to the hospital at 37 and 38 weeks of pregnancy respectively, were treated after delivery.

### Clinical characteristics

No patients were vaccinated for SARS-CoV-2. The median BMI was 24.8 kg/m^2^ (IQR 21.62–27.62, range 21.3–30.5). The median number of comorbidities was 1 (IQR 0.25–1, range 0–3). The most prevalent comorbidity was weight excess defined by a BMI > 25 kg/m^2^ (4 patients). Two patients were obese (BMI > 30 kg/m^2^). All patients presented at least one COVID-19 related symptom, six patients had moderate COVID-19 at the time of mAbs administration, four had severe disease. Variant data were available for nine of the ten patients, and they all were deemed to have the B.1.617.2 variant.

### Maternal treatments and outcomes

All the patients received casirivimab/imdevimab. Two outpatients and three inpatients with mild symptoms hospitalized for reasons unrelated to COVID-19 (two were hospitalized for giving birth and found to be SARS-CoV-2 positive, one was hospitalized for severe stypsis) received the 2.4 g dose treatment. Five inpatients seronegative for anti-Spike IgG at admission received the 8 g dosage. The treatment was started after a median of 2 days from symptoms onset (range 2–11, IQR 2.25–6.25).

No patient had adverse effects related to the use of mAbs. Only symptomatic treatment with ibuprofen was recommended for outpatients. These patients quickly recovered without any complications.

All inpatients received antithrombotic prophylaxis with enoxaparin for the duration of admission. Among inpatients, four required low flow oxygen via mask/nasal cannulae. Three inpatients were treated with steroids (two received dexamethasone 6 mg/die, one 6-methylprednisolone 1 mg/kg) in addition to mAbs. None of the patients received remdesivir. According to the Italian recommendations, seven patients were not eligible for remdesivir treatment (6 did not require oxygen therapy and 1 presented more than 10 days after the onset of symptoms). For the remaining three patients this treatment was not considered by the managing physician. One woman (Table 1, patient 7) hospitalized to give birth was treated with 2.4 g casirivimab/imdevimab. She developed a severe respiratory failure, requiring a transient escalation to high flow oxygen and non-invasive ventilation, but was finally discharged without oxygen support. One patient developed myocarditis, attributed to COVID19 disease complication. No patient required intensive care unit (ICU) admission, and none died. All hospitalized patients were discharged without oxygen support. Among the eight inpatients, the median hospitalization duration was 7 days (range 4–10, IQR 4.75–9.25).

**Table 1:** Feature of pregnant and postpartum women treated with the monoclonal antibodies (mAbs) casirivimab/imdevimab, Careggi University and Hospital, Florence, Italy, March to September 2021

Patient n	Age (years)	Gestational age at mAbs treatment (weeks)	Days between COVID-19 symptoms onset and monoclonal antibodies administration	Inpatient (I)/outpatient (O)	Hospitalization days	Administer Casirivimab/imdevimab dose	Pregnancy and neonatal outcome	NIH severity scale at monoclonal antibodies administration^a^	Body Mass Index (kg/m^2^)	Additional risk factors for severe COVID-19^b^	O_2_ therapy	Steroids
1	29	23	4	I^c^	4	2.4 g	Full term pregnancy. Complicated vaginal delivery leading to transitory admission of the neonate in pediatric ICU	Moderate	22	None	No	No
2	30	24	4	O	0	2.4 g	Uneventful	Moderate	24	None	No	No
3	37	17	11	I	10	8 g	Uneventful	Severe	25.6	Cardiac disease	Yes	Yes
4	30	Postpartum	2	I	7	2.4 g	Uneventful	Moderate	26.5	Overweight	No	No
5	30	24	7	O	0	2.4 g	PROM^d^ at 34 + 4 w, vaginal delivery. Neonate premature but asymptomatic	Moderate	30.5	Obese	No	No
6	29	17	2	I	4	8 g	Uneventful	Moderate	28	Overweight	No	No
7	32	Postpartum	2	I	11	2.4 g	Uneventful	Severe	30.5	Obese	Yes	Yes
8	32	30	4	I	9	8 g	Pre-eclampsia, urgent c-section at 36 w complicated with bleeding and anemia. No neonatal sufferance	Moderate	21.3	Substance abuse, Smoking, Mental Health issue	No	No
9	34	24	3	I	5	8 g	Fetal pathological cardiotocographic trace, urgent c-section at 38 w. Neonate asymptomatic	Severe	21.3	None	Yes	No
10	38	32	9	I	7	8 g	Vaginal delivery at 39 + 3 w complicated with post-partum bleeding. Mild and transient neonatal jaundice	Severe	21.5	None	Yes	Yes

^a^The National Institutes of Health (NIH) scale was used to define COVID-19 severity (https://www.covid19treatmentguidelines.nih.gov)

^b^Risk factors for severe COVID-19 were defined according to the CDC classification (https://www.cdc.gov/coronavirus/2019-ncov/need-extra-precautions/people-with-medical-conditions.html)

^c^Patient was hospitalized for severe stypsis

^d^Premature rupture of membranes

At a 28 days follow-up telephone call, all patients were clinically recovered, and seven patients had performed a negative molecular nasopharyngeal swab for SARS-CoV-2.

### Neonatal/fetal outcomes

At the time of this writing, all women included in the study have already delivered. Five patients had uncomplicated deliveries. Two women had a premature delivery, one because of premature rupture of membranes premature at 34^+4^ weeks of pregnancy, one because of pre-eclampsia at 36 weeks of pregnancy requiring urgent caesarean section which was complicated by bleeding and anemia. Another woman required caesarean section because of fetal pathological cardiotocographic trace; however, the neonate was asymptomatic. One woman presented with mild postpartum bleeding. One newborn required transitory admission in the pediatric ICU and another developed mild and transient neonatal jaundice. None of the above events were deemed related to the mAbs treatment considering the timing of mAbs administration and the onset of the event. All women were allowed to breastfeed.

Table 1 summarizes the information on the study patients.

## Discussion

Recently some reports on pregnant women treated with mAbs have become available, the majority of whom do not report concerning adverse effect and good efficacy [23–27]. This is not a surprise, as immunoglobulins in pregnancy have been largely used to prevent haemolyses caused by maternal/foetal RhD-incompatibility [28]. Moreover, several other monoclonal antibodies are used in pregnant patients [29]. For instance, the NIH guidelines allowed the use of tocilizumab in pregnant patients, stating that pregnant patients with COVID-19 should be treated with the same strategies used for other patients [14]. Our report has several limitations: firstly, we included both patients treated during pregnancy and patients treated in the postpartum. In fact, we considered that patients in the very first days following delivery were still at increased risk of developing severe COVID19, since the vulnerability induced by pregnancy do not immediately recede after delivery [4, 5, 15]. Of note, the patient who required the highest level of care was among those treated after delivery.

Our patient population was demographically similar to other cohorts of pregnant patients with COVID-19 [19]. Moreover, consistently with the epidemiological scenario at the time of mAb administration [30] most of our patients presented with the B.1.617.2 variant. Other studies have also noted how pregnant patients with COVID-19 tend to be relatively healthy [31], which supported the role of pregnancy as a predisposing factor for SARS-CoV-2 infection and severe disease manifestations. Recent advances in pregnancy immunology suggest that pregnancy begins in a pro-inflammatory environment that allows implantation and placentation, then shifts to an anti-inflammatory stage that allows fetal growth, and finally shifts back to a pro-inflammatory stage that promotes labour [32]. The immunological basis underlying pregnant women predisposition to severe COVID-19 is not fully clear. Some authors suggested that the pregnancy induced modifications in Th17 regulation—a critical feat of immunomodulation [33]—which could be the basis for an increased inflammatory response to SARS-CoV-2 infection in these patients.

Moreover, while Italian recommendations on the use of mAbs do not explicitly list pregnancy as a risk factor for severe COVID-19, they include secondary immunodepression as eligibility criteria for mAbs in the outpatient setting, allowing the use of casirivimab/imdevimab in pregnant patients, as also suggested by the NIH recommendations [14].

Of course, the small sample of patients and the lack of a control group in our study do not make general conclusions about the efficacy and safety of mAbs against SARS-CoV-2 in this special population. However, we found that after mAbs administration, most patients did not progress to a severe disease. This is particularly relevant as two meta-analyses showed that the use of maternal ventilation (including C-PAP or mechanical ventilation) worsened the prognosis for both mother and foetus and increased the incidence of pre-term delivery in mother with COVID-19 [6, 34]. Moreover, the use of mAbs does not exclude the use of other drugs currently employed as standard treatment for COVID-19. Some patients in our cohort were treated with steroids due to the presence of pneumonia and hypoxia, like in the RECOVERY trial arms that tested the use of casirivimab/imdevimab 8 gr on inpatients [12].

On the other hand, our experience with outpatients also suggests that casirivimab/imdevimab can be safely used in pregnant inpatients in the early phase of disease since we did not observe mAbs related side effects.

The current lack of data should prompt other authors to share their experience with these compounds in pregnant women. This population is often neglected in clinical studies due to the risk connected with testing new preventive measures or treatment on pregnant women: this once again became true when COVID-19 vaccine trials were conducted [8, 9], leading to a delay in the start of immunization campaigns for pregnant women. This is even more important since recent data indicate that the risk for severe COVID-19 increases with gestational age [35].

During the editorial process for this paper, the rise and spread of the Omicron variant of concern has likely rendered the compound used in this case series useless for patients with COVID-19 in areas where this variant is dominant due to the high rate of mutations present in the receptor-binding domain [36]. For this reason, the NIH guidelines were updated to recommend against the administration of casirivimab/imdevimab if Omicron is found to be circulating at a prevalence of over 80% [14]. Another mAb, sotrovimab, seems to retain efficacy in vitro, but further studies are needed to clarify this in a real life setting [36]. Recently two oral drugs (molnupiravir and nirmatrelvir/ritonavir) became available to treat mild to moderate COVID-19 and data on 3-day early treatment with intravenous remdesivir were published. These drugs are expected to retain activity against the Omicron variant [14]. However, because remdesivir requires intravenous infusion for 3 consecutive days, there may be logistical constraints to administering it in many settings and the oral drugs are not recommended during pregnancy and breastfeeding. For this reason, in the “Omicron era” pregnant and postpartum women could become a target population that may benefit from last generation mAbs, such as sotrovimab, since oral treatment options are contraindicated.

In conclusion, our experience suggests that mAbs against SARS-CoV-2 can be a safe tool to treat pregnant and postpartum patients in the first phases of the disease, or seronegative patients hospitalized for COVID-19. Further studies are needed to assess better the magnitude of the impact on patient morbidity and mortality.
